# Macon Medical Society

**Published:** 1894-02

**Authors:** K. P. Moore

**Affiliations:** Reporter; Macon, Ga.


### MACON MEDICAL SOCIETY.

MACON, GA., December 5th, 1893.

*Dr. A. Blain, President, Presiding.*

It being the evening for the Section on Gynecology to report, and Dr. K. P. Moore being the only member of the section present, he was invited by the chair to make a rêport. Dr. Moore stated that as he had not been notified that it would be the time for his section to report, he had not formulated or prepared a report, but would, in a desultory manner, report two operations, the result of which had been very gratifying. About the 10th of last April went to Smithville to do an ovariotomy for a patient of Drs. Simpson & Clarke. Found a large uterine fibroid, with uterus extending to umbilicus. Opened abdominal cavity with a view of removing uterus, tubes and ovaries, if practicable. Found uterus filling the pelvic cavity, with extensive adhesions, and with but slight mobility of the organ. Finding it entirely impracticable to undertake the removal of this entire mass, removed the tubes and ovaries, and closed up the cavity, trusting to nature to take care of the fibroid growth.

Patient made a good recovery from the operation, and the tumor has gradually subsided, and patient's general condition has greatly improved and she now gives promise of making a complete recovery and taking a new lease on life. She is an unmarried woman, twenty-eight or thirty years of age, but has not menstruated since the operation. An aunt of the patient, who was seen a few days ago, reported that the tumor had diminished so greatly that it was now scarcely noticeable.

Dr. Ferguson asked Dr. Moore if there was no way of curing these patients other than by operation.

Dr. Moore replied that it would depend upon the character of the growth. If it was a sub-mucous fibroid of recent origin, and pointed into the uterine cavity it could usually be removed by operation through the cervical canal, particularly so if it was at all pedunculated, and even if it was not. We can often make it pedunculated by applying the ecraseur wire close to the base of the tumor just where it emerged from the uterine wall. He had recently operated in this way upon three cases of sub-mucous fibroids of recent growth with perfect success. But if the growth was mural or sub-peritoneal, there was not much to be hoped for from medicines, and the ovariotomy gave better results than any other plan. Apostoli and others had accomplished some brilliant results by the use of electricity, and it might be that we could all accomplish more by this method if we understood it better. Ergot, the iodines, ammonias and potashes have all been weighed in the balance and found wanting, and the operation of ovariotomy has given better results than any other remedy in those cases which cannot be enucleated through the cervix.

DR. MCHATTON reported a case of mural fibroid that had been treated with the persistent use of ergot that had died from septicemia, and the post mortem revealed a small necrosed tumor that had been pressed upon by the circular fibers of the uterus, under the influence of the ergot, and from this source of infection she suffered blood poisoning and death. Dr. Ferguson said that he had always cured uterine fibroid by exhibiting for some time, saturated solution of mur. ammonia or chlorate potash, and he believed that a cure could always be had in this way.

The other case reported by Dr. Moore was that of peri-uterine abscess, starting perhaps as an original pyosalpinx, but which now involved the tube and ovary and connective tissue of the left side and pointed into the rectum. When patient was first seen, fourteen months ago, a laparotomy was advised, but was declined by patient's husband. The abscess cavity was then aspirated through the rectum with an Allen pump and washed out. For a time patient seemed to do well and gave promise of recovery, but following the usual history of such cases the abscess refilled, and after several weeks discharged itself through the uterus. This was repeated twice or three times during the year following the formation.

After the patient became a wreck and her life was despaired of the obstinate husband relented, and an operation was sought by wife and husband. Patient was taken into Dr. Moore's Infirmary about the first of October, 1893, and with a forlorn hope and little or no prospect of success, the belly was opened. The whole of left pelvic cavity was found to be filled with adventitious growth, more or less cicatricial, involving tube, ovary, rectum and uterus with deep fluctuation. It was decided that to undertake the removal of this mass would involve an opening into the colon and rectum and require such extensive separation of tissue and probable hemorrhage as to leave little or no hope for the patient in her emaciated condition to survive the operation. It was found to be entirely practicable to make an incision into this abscess cavity from the vagina, and by gentle pressure on this mass from within it was made sufficiently pointing into the vagina to be freely opened, which was now done, and the abdominal cavity was sponged out and the incision closed. Before the patient recovered from the ether a speculum was introduced, the opening was made large and free, the abscess cavity washed out and packed with iodoform gauze. The recovery from the operation was everything that could have been wished. The vaginal incision has been kept open and the cavity washed and repacked every other day. The pelvic mass has gradually melted away, the uterus has become quite movable, the uterine inflammation has subsided, the patient has gained strength and flesh, her complexion cleared up and she is now able to be about the house, goes to the table and is ready for three square meals per day. The abscess cavity is gradually filling up by granulation, and this patient also promises to take on a new lease of life.

Dr. H. J. Williams read a letter from one of the Medical Directors of the Penn Mutual Life Insurance Company, asking as to whether or not it was true that the average Southern women menstruated beyond the ordinary age for the menopause. The writer said that death claims from the South for women dying from miscarriages or childbirth over the average age for the cessation of the catamenia were larger than from other sections of the country. Not infrequently these claims came from women at fifty years old and over.

Dr. Williams had known of several women who menstruated up to the age of fifty and over and one as old as fifty-nine.

Dr. Gaston had known several instances of women menstruating who were over fifty years of age, and mentions a case of birth at fifty-two.

Dr. Moore said that while he had never thought of this subject as applicable especially to Southern women, yet he thought that it not infrequently happened that our Southern women menstruated up to fifty years of age and over, and that occasionally they bore children up to that age. He mentioned having delivered one of a healthy child at fifty-four years old, mention of which was made in a paper read by him before the Georgia Medical Association several years ago.

Dr. McHatton thought it was true that as we approached the tropics women began to menstruate earlier, and ceased to menstruate earlier.

Dr. Gilmer thinks that the principle is well established that where menstruation came on early it usually lasted until late in life, and while it was true that as we approach the tropics menstruation came on earlier, it was also true that it continued later, and hence he could see how Southern women menstruated longer than the Northern or Eastern women.

Macon Medical Society met December 19. Dr. Jas. T. Ross, Vice-President, in the chair.

This being the time for the report of the Section on Surgery, its Chairman, Dr. E. G. Ferguson, stated that Dr. H. J. Williams had prepared and expected to read an essay on "Amputation and Dressings," but he had been called out of the city, and had not left his manuscript with any member of the section. In lieu of a formal report, Dr. Ferguson reported the case of a child, fourteen months old, in whom a small tumor was discovered in the left side just under the margin of the ribs, fifteen weeks ago. It had grown rapidly, and a few days ago proved fatal. He had made a post mortem and now had the pleasure to present the pathological specimen, an immense fatty tumor, weighing six and three-fourths pounds. The spleen was loosely attached to the tumor but was not enlarged, nor especially diseased. The tumor was sub-peritoneal, but filled the entire abdominal cavity. The kidney was normal in size and situation, except slightly flattened from pressure. The attachments were very slight and easily broken up; he had no trouble in lifting it out and removing it. There was no special point of attachment like a pedicle. During the last several weeks Drs. Williams, Gilmer, Derry, Blain, Worsham and Moore had seen the case and none of them had made a correct diagnosis of the case, most of them diagnosing cysts, some of spleen and some of kidney. A trocar had been passed into the tumor at two different times with negative results. Drs. Moore and Blain, when they saw the patient, were in faver of an exploratory laparotomy and if possible the removal of the tumor. Dr. Ferguson now believes that an operation at the time it was proposed by Dr. Moore might have resulted in saving the life of the patient.

At the last meeting of the Society, when "Amputations and their Dressings" was announced as the subject for this meeting, the President appointed Drs. Moore, Gibson and Blain to discuss the subject, and the President called upon Dr. Moore to lead in the discussion.

DR. MOORE said as he had anticipated great pleasure in hearing the subject exhaustively and ably treated by the essayist, Dr. Williams, he had made no preparation, and would have to make his remarks in a desultory way, and upon general principles.

He knew of nothing new, either in amputation or dressings. The fundamental principles of amputations, and the technique of all amputations have been settled years ago, and there seems now nothing left to discuss on this line. Wyeth's hip-joint amputation was perhaps the last forward step on the line of this class of work, and that has now become an established and well-recognized orthodox operation. And the question of well-regulated antiseptic dressings, thoroughly guarding the wound against the possible invasion of germs, has become so well established as to make any man guilty of criminal neglect who fails to avail himself of them. And so there seems to be almost nothing left to discuss on the subject of "amputations and their dressings." There is one point which does not seem quite settled yet; at least there is some difference of opinion of drainage. In my own opinion I think there is no question in the whole range of the full technique of an amputation which is more fully settled than that of proper drainage; and should I be the judge, I should say that few, if any, of the major amputations would be complete without proper drainage, the opinion of some good operators to the contrary notwithstanding. To illustrate, sometime ago I made an amputation of the breast. The dissection was so clean, and the flaps so perfectly coaptated and fitted the cut surface so perfectly, I felt that all that was necessary was to place a well-fitting pad over the whole face of the field of operation, with properly adjusted smooth bandage, and I felt certain of getting union by first intention of the whole surface. The edge of my wound soon became agglutinated, the natural oozing from the wound was pent up in the cavity from which the breast had been amputated, and, to my mortification, I was forced to take down my dressing in a few days to find the whole thing ballooned up full of muddy, wine-colored serum, all of which would have been obviated by keeping the wound drained for forty-eight or seventy-two hours. And so I think that no matter how promising a wound we may have, it is always good surgery to provide for the natural oozing, which will always result from any extensive cut surface. I would caution against leaving the drainage tube, or whatever may be used, in too long; usually forty-eight or seventy-two hours is sufficient for most wounds. Of course the varying circumstances will indicate the length of time to leave the drainage. One form of dressing I would suggest for almost all amputations or other incisions, and that is wet cotton covering over the wound, wet in a solution of mercury bichloride and squeezed as dry as can be. I use this always in all closed wounds instead of iodoform or bichlor gauze. In all my abdominal work I place immediately over the closed incision a pad of this dressing and then a large pad of dry cotton and over all this the flannel binding; and so I do over all wounds where the skin is brought together and where there is no risk of absorption. I make a clean, well-fitting dressing, free from any risk and objectionable odor, and effectually bar out all germs.

DR. GIBSON, who was next invited to discuss the subject, said he nad nothing new to add to what had been said by Dr. Moore. He wished to present a patient treated by himself some two months ago, the results of which had been very gratifying to himself as well as the patient. He presented a negro man who had been caught in the band of a large wheel and carried over the upper pulley and shaft of large machinery, ten feet or more high, and which resulted in a fracture of two ribs, a fracture of tibia and fibula at lower end, and dislocation of ankle, and dislocation of shoulder, and a compound comminuted fracture of both bones of left arm in two places. The ends of both bones protruded and the soft parts of the whole arm were badly lacerated and almost literally made into sausage meat. All the fractures and dislocations were now well, and the man had a fair use of the arm. Pronation and supination were tolerably well preserved in that arm and gave promise of a very fair use of the arm. The doctor had pursued the most approved antiseptic dressing in this case. He had used the bookbinder's board in this case as splints, and was largely in the habit of using this form of splints in all his fractures.

Dr. Moore congratulated Dr. Gibson on the result of his skillful management of this case, and spoke of how well this case illustrated modern conservative surgery. He said that in former times, even since he had been in the profession, twenty-five years ago, this patient would have been turned out to earn his living as best he could with one arm. The old surgeons of thirty or forty years ago would have raised their hands in holy horror at any attempt to save this man's mutilated arm, and, perhaps, in those days when listerism was unknown, it would have been a grave risk of septic poisoning to have left that arm to suppurate and "mortify," as it would have probably done.

Most gracious results can now be obtained and many useful members saved by modern methods, that formerly were sacrificed, and the patient sent away maimed for life.

Dr. Ferguson reported a case treated by himself and the late Dr. Blain, at Brunswick, fifteen or twenty years ago, the results of which had been most extremely gratifying and satisfactory, and claimed himself to have been among the pioneers in antiseptic and conservative surgery.

K. P. MOORE, Reporter.

